# Beneficial Effect of Combining Radiotherapy and Transarterial Chemoembolization on Patient Survival in Hepatocellular Carcinomas and Macrovascular Invasion Treated with Sorafenib

**DOI:** 10.3390/cancers15102687

**Published:** 2023-05-10

**Authors:** Meng-Chuan Lu, Wen-Yen Huang, Hsiu-Lung Fan, Teng-Wei Chen, Wei-Chou Chang, Hsuan-Hwai Lin, Yu-Lueng Shih, Tsai-Yuan Hsieh, Wei-Chen Huang

**Affiliations:** 1 Division of Gastroenterology, Department of Internal Medicine, Tri-Service General Hospital, National Defense Medical Center, Taipei 11490, Taiwan; 2 Department of Radiation Oncology, Tri-Service General Hospital, National Defense Medical Center, Taipei 11490, Taiwan; 3 Division of Organ Transplantation Surgery, Department of Surgery, Tri-Service General Hospital, National Defense Medical Center, Taipei 11490, Taiwan; 4 Department of Radiology, Tri-Service General Hospital, National Defense Medical Center, Taipei 11490, Taiwan; 5 Gastrointestinal Unit, Massachusetts General Hospital, Harvard Medical School, Warren 1019A, 55 Fruit Street, Boston, MA 02114, USA

**Keywords:** radiotherapy, transarterial chemoembolization, sorafenib, hepatocellular carcinoma, macrovascular invasion, survival

## Abstract

**Simple Summary:**

Systemic therapy is current standard treatment for patients with hepatocellular carcinoma (HCC) with macrovascular invasion (MaVI). However, the outcome is poor. In the present study, we analyzed the outcome of patients treated with combined sorafenib, radiotherapy (RT), and transarterial chemoembolization (TACE) or sorafenib alone. The result showed superior overall survival in the combined modality group. Moreover, we conducted propensity score matching and multivariable analysis, showing that combined modality resulted in superior overall survival. Thus, we concluded adding TACE and RT to sorafenib might prolong survival in patients with HCC and MaVI.

**Abstract:**

Background: Approximately 10–40% of hepatocellular carcinoma (HCC) patients have definite vascular invasion at the time of diagnosis. Without curative treatment options, these patients have an abysmal prognosis with a median survival of only a few months following systemic therapy. However, supportive evidence of combining multiple locoregional treatments with systemic therapy is limited. This study compared the outcomes of sorafenib alone versus multimodality therapy with sorafenib, radiotherapy (RT), and transarterial chemoembolization (TACE) in advanced HCC patients with macrovascular invasion (MaVI). Methods: The process took place over a nine-year period between March 2009 and October 2017, wherein 78 HCC patients with MaVI who underwent either sorafenib therapy alone (n = 49) or combined sorafenib/RT/TACE (n = 29) therapy were chosen for the retrospective study. We compared the overall survival (OS) between the two groups using the Cox regression hazard model and adjusted imbalances using propensity score matching (PSM). Results: At the last follow-up, 76 patients had died, with a median follow-up time of 4.8 months for all patients and 31 months for those who were alive. Patients treated with sorafenib/RT/TACE had superior OS compared to those treated with sorafenib alone, showing a median survival of 9.3 vs. 2.7 months and a one-year survival of 37.1% vs. 6.1% (*p* < 0.001). In the multivariable analysis, new diagnosis or recurrence of HCC and treatment modalities (sorafenib alone vs. sorafenib/RT/TACE) were independent prognostic factors for OS. Compared to patients treated with sorafenib alone, significantly better OS was further verified using PSM (*p* < 0.001) in patients who received multiple therapeutic modalities. Conclusion: Multimodality therapy with sorafenib/RT/TACE increased OS threefold versus sorafenib therapy alone in HCC patients with MaVI. This study offers promising benefits of combined locoregional and systemic therapy for advanced HCC in current patient management and prospective clinical trials.

## 1. Introduction

Hepatocellular carcinoma (HCC) is the fourth most common cause of cancer-related death worldwide and accounts for approximately 90% of primary liver cancers [1,2]. Macrovascular invasion (MaVI), defined as evident tumor invasion of hepatic, portal vessels, and/or inferior vena cava (IVC) [3,4,5,6], accompanies HCC diagnosis in 10–40% of patients [7,8]. These patients, ineligible for curative surgery, radiofrequency ablation, and liver transplantation, usually have an abysmal prognosis owing to rapid tumor progression or deteriorated liver function.

Since it is beyond locoregional treatment, systemic therapy, such as targeted therapy and/or immuno-oncology (IO) therapy, is the standard treatment for advanced HCC with MaVI under current guidelines [9]. However, according to studies, the response rate was approximately 11.9–27.3%, with the overall survival (OS) rate at 12 months being 54.6–67.2%, and the median progression-free survival (PFS) was approximately 4.3–6.8 months [10,11,12,13,14]. Therefore, increasing treatment responses is urgently needed for these patients.

Sorafenib is a tyrosine kinase inhibitor (TKI) and the first recommended target therapy to improve survival in patients with advanced HCC. It remains one of the first-line systemic treatments for advanced HCC in most clinical guidelines and is still widely used in many countries due to its cost and availability [15,16]. Nevertheless, an unsatisfactory HCC response to sorafenib has been reported in previous research, and the improvement in outcome is rather limited [11,12,13]. Thus, for efficacy enhancement, many clinical studies have tried to combine different modalities of treatments, such as targeted therapy, transarterial chemoembolization (TACE), radiotherapy (RT), or IO therapy [4,5,6,17,18,19,20].

Due to the lack of prospective clinical trials and insufficient evidence to support additional benefits of multi-locoregional therapy for advanced HCC patients treated with sorafenib, we investigated a total of 172 HCC patients with MaVI from 2009 to 2017 in our medical center.

## 2. Materials and Methods

### 2.1. Patients

The study was performed as a retrospective investigation in a tertiary medical center. The medical records of patients with advanced-stage HCC with MaVI who received sorafenib were traced from March 2009 to October 2017. During that time, a multidisciplinary team discussed most of the cases regarding therapeutic plans. This team comprised medical oncologists, radiation oncologists, radiologists, pathologists, hepatologists, and hepatobiliary surgeons.

The inclusion criteria for the patients of this retrospective analysis were as follows: (1) age ≥ 18 years; (2) diagnosis of HCC based on either pathological findings or imaging criteria [21]; (3) macroscopic vascular invasion of the portal vein or inferior vena cava detected by the imaging study of computed tomography (CT) or magnetic resonance imaging (MRI); (4) Child–Turcotte–Pugh (CTP) class A–B liver function; (5) Eastern Cooperative Oncology Group (ECOG) performance status score of 0–3; and (6) receiving either sorafenib alone or a combined therapy modality, defined as sorafenib, radiotherapy, and TACE within two months.

We included both newly diagnosed and recurrent tumors. Recurrent tumors were defined as not de novo disease. To put it another way, the disease was categorized as recurrent disease if the patients had received any previous treatment for HCC. Patients with vascular invasion in the segmental portal vein or hepatic vein and patients who received liver transplantation before or adjuvant use of sorafenib after lobectomy were excluded.

### 2.2. Sorafenib

Sorafenib was administered to both groups of patients at doses of 400–800 mg daily. In general, patients continued sorafenib for as long as possible until disease progression or severe adverse effects occurred, such as grade 3 hand–foot syndrome, uncontrolled hypertension, deterioration of liver function, or elevated serum bilirubin levels.

### 2.3. TACE Procedure

Details of the TACE preparation, technique, and procedures were described in one recent study [22]. In brief, the procedure of selective digital subtraction angiography of the superior mesenteric artery and celiac artery was performed via injection of iopromide (Ultravist 300), followed by injection into the common, right, or left hepatic artery. Two experienced gastrointestinal interventional radiologists determined the feeding arteries of the tumors. In some cases, cone-beam C-arm CT scans were performed with poorly defined tumor-feeding vessels to prove tumor enhancement in the perfusion area. Thereafter, tumor embolization was operated using conventional TACE or drug-eluting microspheres. Furthermore, embolization might not have been conducted due to the potential risk of hepatic failure in some cases with severe portal blood flow impairment at the discretion of interventional radiologists. Repeated or staged TACE procedures were performed for large or multiple lesions. The HCC multidisciplinary team usually determined the courses and necessity of TACE.

### 2.4. Radiotherapy

Radiotherapy was performed using hypofractionated RT or stereotactic body RT (SBRT). The gross tumor volume was a radiographically visible tumor based on contrast enhancement on CT or MRI. The clinical target volume was the same as gross tumor volume (macrovascular tumor thrombosis +/− the connecting intrahepatic tumors). The planning target volume was obtained by adding 0–8 mm expansion to the corresponding clinical target volume; this would be modified when the dose-limiting organs overlapped, except for the normal liver. In SBRT, treatments were performed in 4–5 fractions, with a total dose of 28–60 Gy (median, 45 Gy) prescribed to the planning target volume. Before June 2017, we used CyberKnife (Accuray, Sunnyvale, CA, USA) with respiratory synchrony to manage respiratory motion. After June 2017, patients were treated using the breath-hold technique from Versa HD with the Active Breathing Coordinator (Elekta AB, Stockholm, Sweden).

For hypofractionated RT, we prescribed a total dose of 37.5 to 52.5 Gy in 15 fractions in a three-week treatment.

### 2.5. Follow-Up and Toxicity Assessment

Patients were followed regularly during periodic clinical evaluation, including detailed history and physical examination, ECOG performance status classification, liver function and serum bilirubin testing, and imaging study of abdominal CT scan or magnetic resonance imaging within three months after the completion of TACE or RT. Three-month follow-up intervals were also arranged after that. Treatment side effects or toxicities were scored according to the Common Terminology Criteria for Adverse Events, version 4.0.

### 2.6. Statistical Analyses

Overall survival was calculated as the time from the initial administration of sorafenib alone or combined therapy with sorafenib, RT, and TACE until death from any cause or the last follow-up. The definition of PFS was the time from the date of sorafenib or combined therapy initiation to the date of disease progression or relapse, which was evaluated based on CT or MRI, death related to disease, or last contact. Kaplan–Meier survival analyses with log-rank tests were used to compare the OS and PFS between the two groups. We used the univariable Cox proportional hazards model to determine the potential prognostic factors of OS by using the log-rank test. Furthermore, those prognostic factors with *p*-value < 0.1 for univariable analysis were included in the multivariable model. Additionally, the method of propensity score matching (PSM) was applied to adjust for potential treatment assignment imbalances, with a ratio of 1:1 (sorafenib alone to combination therapy). The aforementioned statistical analyses were performed using SPSS software version 22 (SPSS Inc., Chicago, IL, USA).

The variables included age, sex, etiology, tumor volume, Seventh American Joint Committee on Cancer stage, Barcelona Clinic Liver Cancer (BCLC) stage, cause of viral hepatitis (hepatitis B virus (HBV) or hepatitis C virus (HCV)), recurrent or newly diagnosed HCC, albumin-bilirubin (ALBI) grade, ECOG performance status, number of tumors, location of macrovascular invasion (Vp4, Vp3, Vp2, IVC), metastasis (M) stage, lymph node (N) stage, and number of prior treatments. In addition, the propensity score matching (PSM) method was also analyzed with the same variables. All tests were two-sided, and statistical significance was defined as *p* < 0.05.

## 3. Results

### 3.1. Patient Characteristics

**Figure 1:**
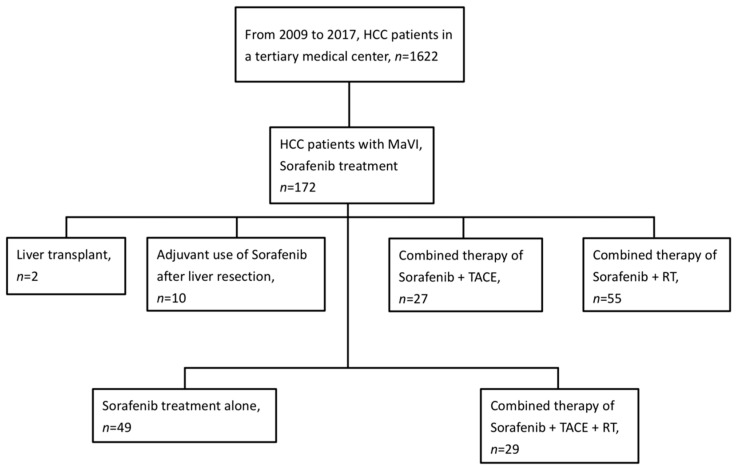
Flowchart of patient inclusion. Abbreviations: HCC= hepatocellular carcinoma; TACE = transarterial chemoembolization; MaVI = macrovascular invasion; RT = radiotherapy.

From June 2009 to August 2017, there were a total of 1622 HCC patients in our medical center. Within that number, our study identified 172 advanced HCC patients with MaVI who had received sorafenib therapy (Figure 1). Ninety-four patients were excluded from our study because of different treatment options undertaken afterward. Eventually, 49 patients treated with sorafenib alone and 29 patients who received multimodal therapy with RT, TACE, and sorafenib were selected for our study (Table 1). At the time of our analysis in 2022, 76 patients had died, and two patients were alive. The follow-up time for live patients was 31 months, despite the median follow-up time of 4.8 months for all our HCC patients. There were 49 (62.8%) patients with CTP class A and 29 (37.2%) patients with CTP class B. There were significant differences between the two groups in age, types of viral hepatitis, recurrence status, prior treatment times, and M stage. After adjustment by propensity score matching, the two matched groups (21 patients each) showed no significant differences in baseline characteristics, except for age and neutrophil-to-lymphocyte ratios (Table 2).

### 3.2. Survival

**Figure 2:**
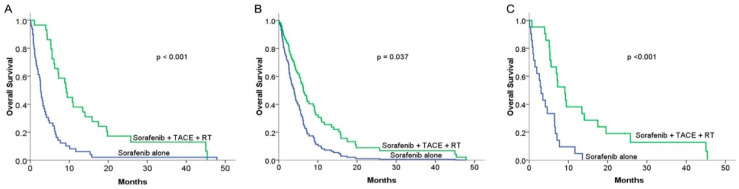
Comparison of overall survival (OS) rates between sorafenib alone and combined therapy groups: (**A**) Kaplan–Meier (KM) curves in the original group (n = 78): median overall survival was 2.83 months and 9.27 months for the sorafenib alone (n = 49) and combined therapy (n = 29) groups, respectively (*p* < 0.001). (**B**) Median OS curve after adjusting for potential prognostic factors using the multivariable Cox regression hazard model in the original group (hazard ratio = 0.52; 95% CI, 0.28–0.96) (*p* = 0.037). (**C**) KM curves of OS in the propensity score matching group (n = 21): the median OS was 3.06 months (95% CI, 1.684–4.436) and 9.11 months (95% CI, 5.97–12.25) for the sorafenib alone and combined therapy groups, respectively (*p* < 0.001).

No treatment-related deaths were reported in either group during the follow-up period. The one-year survival rates according to the Kaplan–Meier curve were 37.1% and 6.1% for patients treated with combined therapy and for patients treated with sorafenib alone, respectively (*p* < 0.001) (Figure 2A). The median OS was 9.3 months in the combination therapy group, which was longer than 2.7 months in the sorafenib alone group (*p* < 0.001). The prognostic factors that could influence OS were analyzed using the Cox proportional hazard model. The overall survival curves of our two groups are shown in Figure 2B (HR = 0.52; 95% CI, 0.28–0.96). In both univariate and multivariate analyses, treatment modalities (combined therapy vs. sorafenib alone (HR = 0.52, *p* = 0.037) and new diagnosis vs. recurrence of HCC (HR = 0.52, *p* = 0.031)) were the factors that significantly affected OS (Table 3). After propensity score matching, combined therapy still showed superior OS than sorafenib alone (*p* < 0.001), with a median survival of 9.11 months (95% CI 5.970–12.250) for the combined therapy group compared with 3.06 months (95% CI, 1.68–4.44) for the sorafenib alone group (Figure 2C). These consistent results of the multivariate analysis and PSM both demonstrated that the treatment modality of combining RT and TACE with sorafenib was superior to sorafenib alone.

### 3.3. Progression-Free Survival

**Figure 3:**
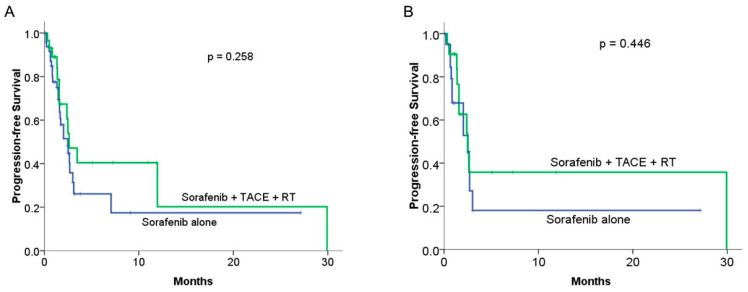
Comparison of median progression-free survival (PFS) between sorafenib alone and combined therapy groups: (**A**) KM curves in the original cohort study (n = 78): median progression-free survival was 2.5 months and 2.63 months for the sorafenib alone and combined therapy groups, respectively (*p* = 0.258). (**B**) The KM curves of PFS in the PSM groups (n = 21 to 21): the median PFS was 2.5 months and 2.56 months for the sorafenib alone and combined therapy groups, respectively (*p* = 0.446).

In comparing PFS between the combined therapy and sorafenib alone groups, the combined therapy did not achieve a significant benefit compared with sorafenib alone, with a median PFS of 2.63 months vs. 2.5 months, respectively (*p* = 0.258; Figure 3A). In the matched cohort, no significant difference in PFS was observed between the two groups, with a median PFS of 2.56 months in the combined therapy group compared with 2.5 months in the sorafenib alone group (*p* = 0.446; Figure 3B).

### 3.4. Toxicity

The most common side effects in both cohorts were fatigue and anemia. The combined therapy group had a higher percentage of leukopenia (grade 1, 34.5% vs. 10.2%), anemia (grade 1, 75.9% vs. 59.2%), and diarrhea (grade 1 and grade 2, 27.5% vs. 14.2%). However, the incidence rates of all skin-related side effects, including hand–foot syndrome, were similar between the two groups. Grade 3 or 4 adverse events related to treatment occurred in 38.8% of the sorafenib alone group and 27.6% of the combined therapy group (Table 4). Grade 5 toxicity or radiation-induced liver disease was not observed in our study patients.

## 4. Discussion

Our research is the first retrospective study to demonstrate survival improvement when combining RT and TACE in advanced HCC patients treated with sorafenib. Regarding OS, either the original HR (0.70, 95% CI, 0.59–0.82; *p* < 0.001) or the adjusted HR using the multivariable Cox regression hazards model (0.52, 95% CI, 0.28–0.96; *p* = 0.037) showed noteworthy improvement in the group of patients who received multimodality therapy. The results indicate that in terms of disease survival, additional locoregional treatments for certain advanced HCCs can contribute a significant benefit over systemic therapy alone.

Sorafenib was the only approved and available systemic therapy for advanced HCC with extrahepatic spread or MaVI during our study. However, according to previous research, the three-month prolongation in OS and the limited response rates of 2–3.3% in HCC patients with MaVI warrant the need for a better treatment combination [11,12]. Indeed, one-year OS was only 6.1% in our sorafenib alone group, even though some patients had relatively favorable conditions and tumor features, allowing for more aggressive treatment combinations at that time.

TACE is generally recognized as one of the standard treatments for unresectable HCC patients [21]. Traditionally, it is not recommended for HCC patients with MaVI to receive TACE due to the increased risk of liver failure [8,23]. However, an increasing number of recent studies show that TACE could still be safely performed in selected patients, provided that there is an adequate hepatic reserve and well-developed periportal collateral circulation around the obstructed portal vein tumor thrombus (PVTT) and good liver function [17,24,25,26]. However, it is usually challenging to achieve a complete response because it has limited efficacy in reducing tumor thrombi, and viable tumor cells may remain after treatment [18].

Aside from TACE, advanced stereotactic body radiation therapy (SBRT) techniques also showed a high local control rate from 87% to 94.6% at two years for unresectable HCC patients [2,27,28,29,30,31]. Another advantage acknowledged by recent research is that, for HCC patients with extensive PVTT initially unsuitable for resection surgery or TACE, RT can achieve both portal vein flow restoration and adequate thrombus shrinkage in the majority of cases [32,33]. Although radiotherapy can be used for locoregional tumor control, its effects are usually partial and relatively temporary [34]. Thus, some studies combined RT with other therapies and have shown that sorafenib given together with RT is a feasible and tolerable treatment option for advanced HCC [34]. One of the possible reasons is that locoregional control from radiotherapy and systemic effects of sorafenib can strengthen overall antitumor efficacy without showing tumor resistance or significant toxicities [30,34]. Moreover, irradiation-induced immune cell priming, such as the so-called “abscopal effect”, for tumor regulation of recurrence or metastasis may also provide another synergistic effect [35,36,37,38].

In fact, patients in better condition and with the expectation of a better prognosis, such as younger age or preserved liver function, are more commonly selected for alternative or combination therapy in real-world clinical practice [39]. Thus, to reduce the bias caused by the uneven allocation of our two groups, we applied two statistical methods, multivariable analysis and PSM, in this study cohort. Our results indeed verified the survival benefits of adding multi-locoregional therapy to systemic therapy, and this should be recommended for all suitable HCC patients with MaVI.

Moreover, our multivariable analysis found that recurrence or new diagnosis of HCC with MaVI was an independent prognostic factor, with a hazard ratio of 0.52, implying a significant impact on patient survival. In line with a recent study, patients who had received previous treatment for HCC exhibited better local control and survival when treated with RT than when treated with TACE. However, no difference was observed between the two modalities among the cohort of patients with newly diagnosed HCC [22]. One explanation for the findings could be that patients with a prior history of HCC may receive inpatient or outpatient follow-up with both laboratory analysis and imaging studies regularly. In that way, those patients are less likely to have delayed detection of tumors in poor general conditions compared to patients with newly diagnosed HCC.

There were no significant differences in PFS between the two groups. One possible reason for this finding is that TACE and RT are considered local treatments, emphasizing vascular invasion with nearby local control. In terms of disease progression, including extrahepatic, lymph node, and intrahepatic metastasis, targeted therapy or immune therapy plays a more crucial role due to their systematic efficacy.

One limitation of this study is that it was a retrospective investigation with difficulty capturing all adverse effects of treatment merely from medical records. In some cases, we could only make determinations using serology data comparison, liver enzyme changes, and records of abdominal symptoms or skin-related complaints during or after the course of therapy. To minimize biases of treatment allocation, we applied PSM to balance possible clinical prognostic factors, although some confounding factors could not be excluded completely. Moreover, up to the time of our study, treatment options for advanced HCC with MaVI were limited, since there was no IO therapy or second-line TKI regimen available, nor was there sufficient evidence to support different treatment modules. Hence, medical decisions for nonsubsidized therapy could only be made by private patients and their specialists. In addition, the definite benefits of combination therapy may be underestimated owing to the patients with a higher percentage of severe MaVI, including Vp3 and Vp4, compared to those treated with sorafenib alone. Finally, advanced HCC patients with MaVI comprise a unique group of the whole HCC population; thus, the sample size of this cohort was relatively small. However, a future study on a greater scale would fortify our results.

Although it has been shown in recent sorafenib studies that applying strict criteria for patient selection or adding second-line therapy of regorafenib for progressed patients can offer more significant prolongation of OS [40,41], therapy with atezolizumab plus bevacizumab is the preferred first-line systemic therapy regimen according to the 2022 BCLC guidelines [9]. Nevertheless, the results of our study can be used as an important clinical reference for current advanced HCC management. Since different treatment modalities have diverse mechanisms to eliminate tumor cells, multi-locoregional therapy together with systemic therapy can contribute additional synergetic effects for patients with advanced HCC and MaVI.

## 5. Conclusions

Overall, the main point of this study is that despite other high-potency TKI and IO therapies being currently available, combining locoregional therapy, such as TACE and RT, should be considered in advanced HCC treatment, and further large-scale prospective randomized trials are warranted.

## Figures and Tables

**Table 1:** Patient characteristics (N = 78).

		Sorafenib Alone	Sorafenib + TACE + RT	*p*-Value
		No. (%)	No. (%)	
No. of patients		49 (62.8)	29 (37.2)	
Sex	Male	34 (69.4)	23 (79.3)	0.340
	Female	15 (30.6)	6 (20.7)	
Age, years	Mean	65	56	0.003
	Median	64	56	
	Min–Max	37–91	33–79	
Viral hepatitis	No	11 (22.4)	1 (3.4)	0.023
	HBV	23 (46.9)	22 (75.9)	
	HCV	15 (30.6)	6 (20.7)	
ECOG	0	6 (12.2)	8 (27.6)	0.055
	1	23 (46.9)	17 (58.6)	
	2	18 (36.7)	3 (10.3)	
	3	2 (4.1)	1 (3.4)	
Recurrent status	New diagnosis	34 (69.4)	3 (10.3)	<0.001
	Recurrence	15 (30.6)	26 (89.7)	
No. of prior treatments	Median	0	2	<0.001
	Min–Max	0–4	0–6	
Largest tumor size, cm	Mean	9.83	9.57	0.868
	Median	9.1	9.0	
	Min–Max	0–23	1–21	
AFP	Mean +/− SD	10,360.3 (16,008.4)	10,240.7 (14,721.0)	0.974
	median	665.0	3597.0	
	IQR	44.7–16,895.3	257.2–16,335.3	
Bilirubin	Mean +/− SD	1.78 (2.21)	1.88 (3.56)	0.878
	median	1.2	1.2	
	IQR	0.8–1.8	0.9–1.7	
Albumin	Mean +/− SD	3.38 (0.54)	3.34 (0.50)	0.788
	median	3.4	3.4	
	IQR	3.0–3.8	3.0–3.7	
INR	Mean +/− SD	1.10 (0.19)	1.08 (0.10)	0.662
	median	1.1	1.1	
	IQR	1.0–1.1	1.0–1.1	
AST	Mean +/− SD	116.4 (125.0)	186.8 (378.0)	0.266
	median	74.0	91.0	
	IQR	48.0–158.5	41.0–148.5	
ALT	Mean +/− SD	83.0 (130.4)	125.2 (274.1)	0.361
	median	42.0	53.0	
	IQR	22.5–83.5	30.5–101.0	
NLR	Mean +/− SD	4. 70 (4.57)	6.37 (3.80)	0.102
	median	3.6	4.8	
	IQR	2.5–4.9	3.1–9.7	
MaVI location	VP2	7 (14.3)	2 (6.9)	0.116
	VP3	21 (42.9)	20 (69.0)	
	VP4	18 (36.7)	7 (24.1)	
	IVC	3 (6.1)	0 (0)	
N stage	0	31 (63.3)	24 (82.8)	0.068
	1	18 (36.7)	5 (17.2)	
M stage	0	35 (71.4)	27 (93.1)	0.022
	1	14 (28.6)	2 (6.9)	
CTP class	A	29 (59.2)	19 (62.1)	0.801
	B	20 (40.8)	11 (37.9)	
ALBI grade	1	6 (12.2)	2 (6.9)	0.518
	2	33 (67.3)	24 (79.3)	
	3	10 (20.4)	4 (13.8)	

AFP, alpha-fetoprotein; ALBI, albumin bilirubin; ALT, alanine aminotransferase; AST, aspartate aminotransferase; CTP, Child–Turcotte–Pugh; ECOG, Eastern Cooperative Oncology; HBV, hepatitis B virus; HCV, hepatitis C virus; INR, international normalized ratio; IQR, interquartile range; M, metastasis; MaVI, macrovascular invasion; N, lymph node; NLR, neutrophil-to-lymphocyte ratio; No, number; RT, radiotherapy; SD, standard deviation; TACE, transarterial chemoembolization.

**Table 2:** Prognostic factors influencing OS using the Cox proportional hazards model (original, N = 78).

	OS			
Variables	Univariable		Multivariable	
	HR (95% CI)	*p*	HR (95% CI)	*p*
Age, years				
≥60 vs. <60	1.22 (0.77–1.92)	0.391		
Sex				
female vs. male	0.83 (0.49–1.41)	0.498		
Etiology				
HBV vs. no	0.59 (0.30–1.14)	0.116		
HCV vs. no	1.10 (0.54–2.25)	0.787		
ECOG		0.005	1.11 (0.84–1.48)	0.459
0–1 vs. 2–3	0.48 (0.29–0.48)			
Status				
recurrence vs. new diagnosis	0.32 (0.20–0.53)	<0.001	0.52 (0.28–0.94)	0.031
Tumor size, cm				
≥9 cm vs. <9 cm	1.15 (0.72–1.82)	0.563		
No of tumors				
single vs. multiple	0.79 (0.47–1.32)	0.370		
AFP				
≥500 vs. <500	1.25 (0.78–2.00)	0.348		
Bilirubin				
≥1.2 vs. <1.2	0.98 (0.62–1.54)	0.924		
Albumin				
≥3.4 vs. <3.4	0.71 (0.45–1.12)	0.141		
INR				
≥1.1 vs. <1.1	1.08 (0.68–1.71)	0.745		
AST				
≥80 vs. <80	1.40 (0.88–2.22)	0.151		
ALT				
≥50 vs. <50	1.09 (0.68–1.73)	0.719		
NLR				
≥3.9 vs. <3.9	1.03 (0.65–1.62)	0.917		
MaVI location				
Vp4 vs. others	1.07 (0.91–1.26)	0.405		
N stage				
0 vs. 1	0.92 (0.71–1.19)	0.517		
M stage				
0 vs. 1	0.81 (0.61–1.08)	0.152		
CTP class				
B vs. A	1.53 (0.95–2.45)	0.078	1.32 (0.79–2.20)	0.295
ALBI grade				
3 vs. 1 and 2	1.58 (0.86–2.90)	0.141		
Treatment				
Sorafenib + TACE + RT vs. sorafenib alone	0.70 (0.59–0.82)	<0.001	0.80 (0.65–0.99)	0.037

AFP, alpha-fetoprotein; ALBI, albumin bilirubin; ALT, alanine aminotransferase; AST, aspartate aminotransferase; CTP, Child–Turcotte–Pugh; ECOG, Eastern Cooperative Oncology; HBV, hepatitis B virus; HCV, hepatitis C virus; INR, international normalized ratio; M, metastasis; MaVI, macrovascular invasion; N, lymph node; NLR, neutrophil-to-lymphocyte ratio; No, number; RT, radiotherapy; TACE, transarterial chemoembolization.

**Table 3:** Patient characteristics after propensity matching (N = 42).

		Sorafenib Alone	Sorafenib + TACE + RT	*p*-Value
		No. (%)	No. (%)	
No. of patients		21 (100)	21 (100)	
Sex	Male	14 (66.7)	16 (76.2)	0.495
	Female	7 (33.3)	5 (23.8)	
Age, years	Mean	63.7	55.2	0.032
	Median	66	53	
	Min–Max	37–82	33–79	
Viral hepatitis	No	5 (23.8)	1 (4.8)	0.101
	HBV	8 (38.1)	14 (66.7)	
	HCV	8 (38.1)	6 (28.6)	
ECOG	0	4 (19.0)	3 (14.36)	0.801
	1	11 (52.4)	14 (66.7)	
	2	5 (23.8)	3 (14.3)	
	3	1 (4.8)	1 (4.8)	
Recurrent status	New diagnosis	8 (38.1)	3 (14.3)	0.079
	Recurrence	13 (61.9)	18 (85.7)	
No. of prior treatments	Median	1	2	0.054
	Min–Max	0–4	0–6	
Largest tumor size, cm	Mean	10.1	9.05	0.516
	Median	8.0	8.0	
	Min–Max	1–23	3–17	
AFP	Mean +/− SD	12,282.5 (16,904.3)	10,661.7 (15,148.2)	0.745
	median	2264	4530.0	
	IQR	40.3–26,711.8	178.5–16,485.3	
Bilirubin	Mean +/− SD	2.38 (3.15)	1.29 (0.56)	0.125
	median	1.4	1.2	
	IQR	0.8–2.2	0.9–1.9	
Albumin	Mean +/− SD	3.38 (0.59)	3.34 (0.54)	0.808
	median	3.4	3.3	
	IQR	3.0–3.9	3.0–3.8	
INR	Mean +/− SD	1.10 (1.1)	1.09 (0.11)	0.779
	median	1.1	1.1	
	IQR	1.0–1.2	1.0–1.1	
AST	Mean +/− SD	104.8 (95.2)	222.8 (441.2)	0.238
	median	74.0	57.0	
	IQR	48.0–103.5	38.5–202.5	
ALT	Mean +/− SD	68.8 (63.7)	143.1 (321.5)	0.305
	median	47.0	44.0	
	IQR	27.5–75.0	27.0–103.5	
NLR	Mean +/− SD	3.83 (2.06)	6.17 (3.63)	0.014
	median	3.6	4.8	
	IQR	2.6–4.9	3.1–9.6	
MaVI location	VP2	4 (19.0)	2 (9.5)	0.607
	VP3	12 (57.1)	12 (57.1)	
	VP4	5 (23.9)	7 (33.3)	
	IVC	0 (0)	0 (0)	
N stage	0	14 (66.7)	16 (76.2)	0.495
	1	7 (33.3)	5 (23.8)	
M stage	0	16 (76.2)	197 (94.5)	0.214
	1	5 (23.8)	2 (9.5)	
CTP class	A	11 (52.4)	13 (61.9)	0.533
	B	10 (47.6)	8 (38.1)	
ALBI grade	1	2 (9.5)	2 (9.5)	0.282
	2	13 (61.9)	17 (81.0)	
	3	6 (28.6)	2 (9.5)	

AFP, alpha-fetoprotein; ALBI, albumin bilirubin; ALT, alanine aminotransferase; AST, aspartate aminotransferase; CTP, Child–Turcotte–Pugh; ECOG, Eastern Cooperative Oncology; HBV, hepatitis B virus; HCV, hepatitis C virus; INR, international normalized ratio; IQR, interquartile range; M, metastasis; MaVI, macrovascular invasion; N, lymph node; NLR, neutrophil-to-lymphocyte ratio; No, number; RT, radiotherapy; SD, standard deviation; TACE, transarterial chemoembolization.

**Table 4:** Toxicity.

	Sorafenib			Sorafenib, RT, and TACE		
	No. of Patients (%)			No. of Patients (%)		
	Grade 1	Grade 2	Grade 3	Grade 1	Grade 2	Grade 3
Leukopenia	5/10.2			10/34.5	2/6.9	
						
Anemia	29/59.2			22/75.9	2/6.9	
						
Thrombocytopenia	16/32.7	3/6.1		9/31	6/20.7	
						
ALT	12/24.5	6/12.2		8/27.6	7/24.1	2/6.9
						
Alk-P	2/4.1	10/20.4		1/3.4		
						
Bilirubin	11/22.4	2/4.1	1/2	8/27.6	6/20.7	
						
r-GT	4/8.2	11/22.4		1/3.4		1/3.47
						
Nausea	6/12.2	3/6.1	9/18.4	4/13.8		
						
Vomiting	2/4.1		2/4.1	1/3.4		
						
Anorexia	32/65.3		9/18.4	9/31		
						
Diarrhea	6/12.2	1/2		5/17.2		
						
Hand–foot syndrome	6/12.2	1/2		2/6.9	3/10.3	
						
Other skin reaction	3/6.1	1/2		5/17.2	1/3.4	1/3.4
						
Fatigue	38/77.6			20/69	1/3.4	
						
Hair loss	1/2					

Alk-P, alkaline phosphatas; ALT, alanine aminotransferase; No., number; r-GT, r-glutamyl transpeptidase; RT, radiotherapy; TACE, transarterial chemoembolization.

## Data Availability

All data generated or analyzed during this study are included in this published article.
